# Supplementary material for: Frequency selective wave beaming in nonreciprocal acoustic phased arrays
Source: Sci Rep. 2020 Dec 7;10:21339. doi: 10.1038/s41598-020-77489-x (PMC7721751; doi:10.1038/s41598-020-77489-x)
Supplement: Supplementary file 1 — Supplementary Information. [file 41598_2020_77489_MOESM1_ESM.pdf]

Supplementary Information: Frequency Selective Wave Beaming in Nonreciprocal Acoustic Phased Arrays

Revant Adlakha[1,+], Mohammadreza Moghaddaszadeh[2,+], Mohammad A. Attarzadeh[1,+], Amjad Aref[2], and Mostafa Nouh[1,*]

[1]Department of Mechanical and Aerospace Engineering, University at Buffalo, Buffalo, NY 14260, USA
[2]Department of Civil, Structural and Environmental Engineering, University at Buffalo, Buffalo, NY 14260, USA
[+]these authors contributed equally to this work
[*]Corresponding author: mnouh@buffalo.edu

1 Scattering Matrix

A conventional acoustic phased array can be considered as a two-port network operating at a frequency ω when the amplitude of the waves is measured at a standard distance away from the array, and at an angle θ_s from the broadside as shown in Fig. S1a. In order to satisfy far-field approximations, we place a pressure sensor (Port 2 in Fig. S1a) at $(r, \theta) = (R, \theta_s)$, with $R = 15\lambda$ in the polar coordinates of the array. As detailed in the main text, an input voltage $\mathcal{V}_{\text{in}} = V_0 e^{i\omega t}$ causes an output pressure amplitude to appear at the pressure sensor and is given by $\mathcal{P}_{\text{out}} = \frac{N}{R}\mathcal{T} V_0 e^{i\omega t}$, where N is the total number of transducers.

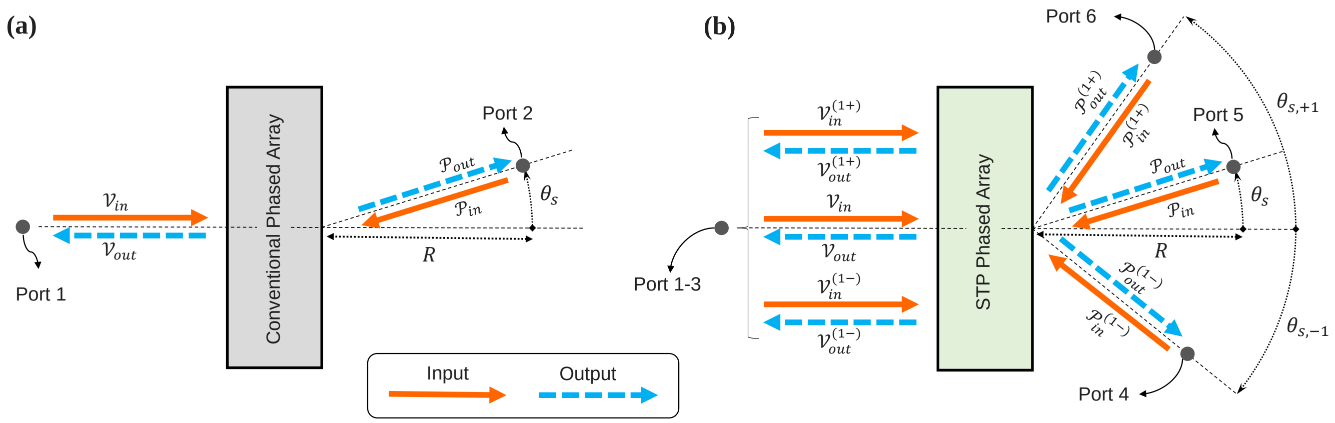

Figure S1. (a) Two-port network model of a conventional phased array showing single channel operation at a frequency ω frequency. (b) Six-port approximate network model of a STP phased array showing three channels of operation at three distinct frequencies ω, $\omega^{(1-)}$, and $\omega^{(1+)}$.

We assert here that the transducers are linear, isotropic, and exhibit a flat frequency response with an electromechanical transformation coefficient of $\mathcal{T}$. Similarly, if an acoustic wave with a frequency $\bar{\omega} = \omega$ passes over the pressure sensor with an amplitude $\mathcal{P}_{\text{in}} = \frac{R}{N}\mathcal{T} V_0 \mathrm{e}^{\mathrm{i}\omega t}$ and is incident on the array, then owing to the reciprocal nature of the transducers, the voltage output becomes equal to $\mathcal{V}_{\text{out}} = V_0 \mathrm{e}^{\mathrm{i}\omega t}$. This can be concluded because the principal listening and transmission directions of a conventional array are coincident, i.e., $\bar{\theta}_s = \theta_s$. For ideal (reflection-free and loss-less) transducers, this behavior is best represented by the following matrix notation

$$\begin{bmatrix} \mathcal{V} \\ \mathcal{P} \end{bmatrix}_{\text{out}} = \begin{bmatrix} 0 & N \\ N & 0 \end{bmatrix} \begin{bmatrix} \frac{\mathcal{T}}{R} & 0 \\ 0 & \frac{1}{\mathcal{T}R} \end{bmatrix} \begin{bmatrix} \mathcal{V} \\ \mathcal{P} \end{bmatrix}_{\text{in}} \tag{S1}$$

or $\mathbf{U}_{\text{out}} = \mathbf{S}.\mathbf{T}.\mathbf{U}_{\text{in}}$, where $\mathbf{S}$ is the well-known scattering matrix (S-matrix), and $\mathbf{T}$ carries the electromechanical transformation coefficients as well as the distance effects (hereafter referred to as the T-matrix). As anticipated, we have $S_{12} = S_{21}$ in the S-matrix; an indication of the reciprocal symmetry of conventional phased arrays.

Unlike its conventional counterpart, considerable frequency conversions take place in an STP phased array. However, by considering $\delta = 1.5$, we can discard high-order Bessel functions and only focus on the zeroth- and first-order terms. As such, to provide a full picture of the STP phased array's performance, we need to represent the system with a six-port model as depicted in Fig. S1b. This enables us to account for the non-negligible energy transfer to higher and lower frequencies by dedicating specific ports to each harmonic—signal or wave—component. We purposefully place the pressure sensors (Ports 4-6 in Fig. S1b) at the principal transmission directions denoted by the $\theta_{s,-1}$, θ_s, and $\theta_{s,+1}$ angles. It is important to note that these three angles are not generally coincident with the principal listening directions of the STP phased array (See Theoretical Background in the main text). Following the same line of thought as that utilized for the conventional phased array, and by carefully employing Eqs. (7) and (14) from the main text, we can write $\mathbf{W}_{\text{out}} = \mathbf{S}.\mathbf{T}.\mathbf{W}_{\text{in}}$, where $\mathbf{W} = [\mathcal{V}^{(1-)}, \mathcal{V}, \mathcal{V}^{(1+)}, \mathcal{P}^{(1-)}, \mathcal{P}, \mathcal{P}^{(1+)}]^{\mathrm{T}}$ while adopting the short-hand notation in the paper (frequency up- or down-conversion by $q\omega_m$ is denoted by $(\bullet)^{(q\pm)}$). The S-matrix is now found to be

$$\mathbf{S} = \begin{bmatrix} 0 & 0 & 0 & B_0^{(1-)}(\theta_{s,-1}) & -\mathrm{i}B_{-1}(\theta_s) & 0 \\ 0 & 0 & 0 & -\mathrm{i}B_{+1}^{(1-)}(\theta_{s,-1}) & B_0(\theta_s) & -\mathrm{i}B_{-1}^{(1+)}(\theta_{s,+1}) \\ 0 & 0 & 0 & 0 & -\mathrm{i}B_{+1}(\theta_s) & B_0^{(1+)}(\theta_{s,+1}) \\ A_0^{(1-)}(\theta_{s,-1}) & -\mathrm{i}A_{-1}(\theta_{s,-1}) & 0 & 0 & 0 & 0 \\ -\mathrm{i}A_{+1}^{(1-)}(\theta_s) & A_0(\theta_s) & -\mathrm{i}A_{-1}^{(1+)}(\theta_s) & 0 & 0 & 0 \\ 0 & -\mathrm{i}A_{+1}(\theta_{s,+1}) & A_0^{(1+)}(\theta_{s,+1}) & 0 & 0 & 0 \end{bmatrix} \tag{S2}$$

where the amplitude coefficients A_0, $A_{\pm1}$, B_0, and $B_{\pm1}$ are defined in the main text. The T-matrix is also rewritten as

$$\mathbf{T} = \frac{1}{R}\begin{bmatrix} \mathcal{T} & 0 & 0 & 0 & 0 & 0 \\ 0 & \mathcal{T} & 0 & 0 & 0 & 0 \\ 0 & 0 & \mathcal{T} & 0 & 0 & 0 \\ 0 & 0 & 0 & \frac{1}{\mathcal{T}} & 0 & 0 \\ 0 & 0 & 0 & 0 & \frac{1}{\mathcal{T}} & 0 \\ 0 & 0 & 0 & 0 & 0 & \frac{1}{\mathcal{T}} \end{bmatrix} \tag{S3}$$

For a non-zero modulation frequency ratio, $\frac{\omega_m}{\omega} \neq 0$, one may convince themselves that the scattering matrix in Eq. S2 can be approximated with the following asymmetric form after substituting for A and B coefficients, and further simplification

$$\mathbf{S} \cong N\begin{bmatrix} 0 & 0 & 0 & 0 & 0 & 0 \\ 0 & 0 & 0 & 0 & J_0 & 0 \\ 0 & 0 & 0 & 0 & 0 & 0 \\ 0 & -\mathrm{i}J_1 & 0 & 0 & 0 & 0 \\ 0 & J_0 & 0 & 0 & 0 & 0 \\ 0 & -\mathrm{i}J_1 & 0 & 0 & 0 & 0 \end{bmatrix} \tag{S4}$$

The asymmetry of the scattering matrix is a necessary and sufficient condition of nonreciprocity.

2 Multi-Channel Operation

As demonstrated in this paper, the STP phased array is able to generate three dominant spherical waves in the $\mathbb{TX}$ mode: fundamental, up- and down-converted at three distinct frequencies ω, $\omega^{(1+)}$, and $\omega^{(1-)}$, respectively. Theses waves travel in the principal transmission directions denoted by θ_s, $\theta_{s,+1}$ and $\theta_{s,-1}$ and can be used to transmit information along three independent channels at the same time. To further elaborate, we consider a general case where these three waves are incident on the phased array simultaneously—possibly after reflecting and bouncing off the three targets that the phased array is tracking. For this analysis, we also consider $\delta = 1.5$. The three principal listening directions of the array in the $\mathbb{RX}$ mode are along the $\bar{\theta}_{s,-1}$, $\bar{\theta}_s$ and $\bar{\theta}_{s,+1}$ angles which can approximately coincide with their respective principle transmission directions for a sufficiently small temporal modulation ratio, i.e., $\frac{\omega_m}{\omega} \ll 1$. The waves incident from the three principle listening directions are incident on the phased array with a frequency $\omega^{(1-)}$ along the $\bar{\theta}_{s,-1}$ direction, with a frequency ω along the $\bar{\theta}_s$ direction, and with a frequency $\omega^{(1+)}$ along the $\bar{\theta}_{s,+1}$ direction, respectively. These three incident waves would each generate three voltage signals. By using Eq. (14) from the main text, and denoting the net collected voltage signals in the $\mathbb{RX}$ mode for the down-converted, fundamental and up-converted waves by $\tilde{v}_{-1}$, $\tilde{v}_0$, and $\tilde{v}_{+1}$, respectively, we find that

$$\tilde{v}_{-1}(t) \cong \bar{V}_0 \left[B_0^{(1-)}(\delta, \bar{\theta}_{s,-1}) e^{i\omega^{(-1)}t} - iB_{+1}^{(1-)}(\delta, \bar{\theta}_{s,-1}) e^{i\omega t} - iB_{-1}^{(1-)}(\delta, \bar{\theta}_{s,-1}) e^{i\omega^{(2-)}t} \right] \tag{S5a}$$

$$\tilde{v}_0(t) \cong \bar{V}_0 \left[B_0(\delta, \bar{\theta}_s) e^{i\omega t} - iB_{+1}(\delta, \bar{\theta}_s) e^{i\omega^{(1+)}t} - iB_{-1}(\delta, \bar{\theta}_s) e^{i\omega^{(1-)}t} \right] \tag{S5b}$$

$$\tilde{v}_{+1}(t) \cong \bar{V}_0 \left[B_0^{(1+)}(\delta, \bar{\theta}_{s,+1}) e^{i\omega^{(+1)}t} - iB_{+1}^{(1+)}(\delta, \bar{\theta}_{s,+1}) e^{i\omega^{(2+)}t} - iB_{-1}^{(1+)}(\delta, \bar{\theta}_{s,+1}) e^{i\omega t} \right] \tag{S5c}$$

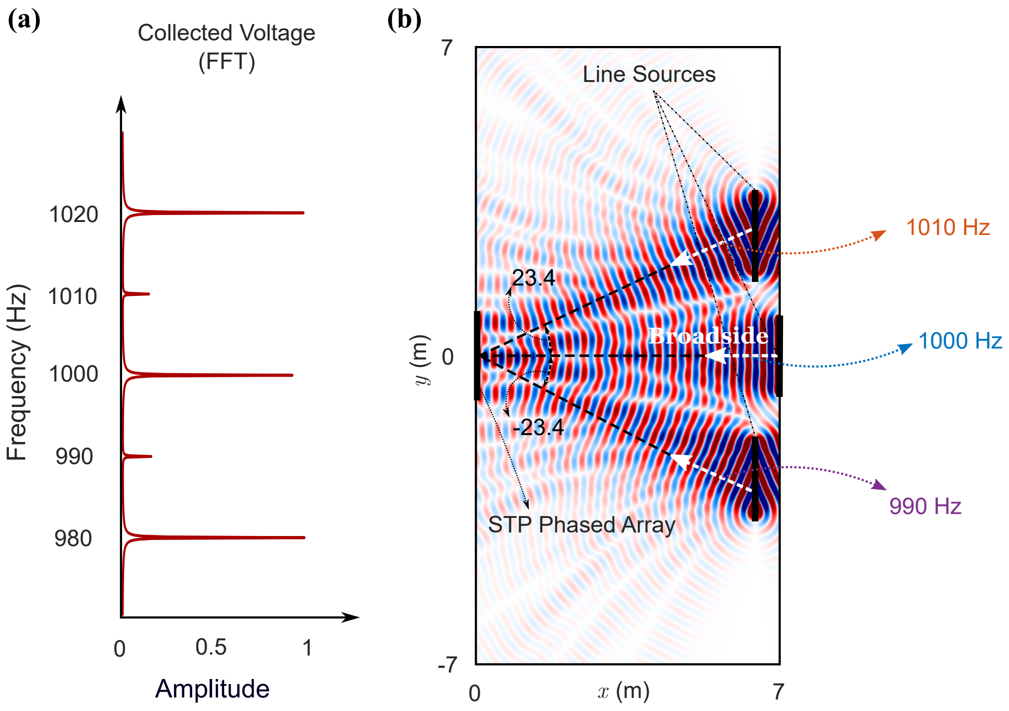

Figure S2. (a) FFT of the collected voltage signal. (b) Time-transient acoustic pressure wave field for simultaneous incidence of three waves at time $t = 1$ second.

From the main text discussion, it is evident that the values of B coefficients are explicit functions of δ and the incident angle $\bar{\theta}$. This implies that for an incident wave along the $\bar{\theta}_{s,-1}$ direction, the amplitude function of B_{-1} is maximum thus indicating the dominance of the frequency $\omega^{(2-)}$ in the collected signal $\tilde{v}_{-1}(t)$. A similar argument can be extended to Eqs. (S5)b and c.

As such, one may approximate Eq. (S5) with

$$\tilde{v}_{-1}(t) \cong -\mathrm{i}\bar{V}_0 B_{-1}^{(1-)}(\delta, \bar{\theta}_{s,-1})\mathrm{e}^{\mathrm{i}\omega^{(2-)}t} \tag{S6a}$$

$$\tilde{v}_0(t) \cong \bar{V}_0 B_0(\delta, \bar{\theta}_s)\mathrm{e}^{\mathrm{i}\omega t} \tag{S6b}$$

$$\tilde{v}_{+1}(t) \cong -\mathrm{i}\bar{V}_0 B_{+1}^{(1+)}(\delta, \bar{\theta}_{s,+1})\mathrm{e}^{\mathrm{i}\omega^{(2+)}t} \tag{S6c}$$

and for simultaneous incidence, the total collected voltage is simply expressed as the sum of the collected voltages, i.e., $\tilde{v}_{\mathrm{tot}}(t) = \tilde{v}_{-1}(t) + \tilde{v}(t) + \tilde{v}_{+1}(t)$, or

$$\tilde{v}_{\mathrm{tot}}(t) \cong \bar{V}_0 \left[B_0(\delta, \bar{\theta}_s)\mathrm{e}^{\mathrm{i}\omega t} - \mathrm{i}B_{+1}^{(1+)}(\delta, \bar{\theta}_{s,+1})\mathrm{e}^{\mathrm{i}\omega^{(2+)}t} - \mathrm{i}B_{-1}^{(1-)}(\delta, \bar{\theta}_{s,-1})\mathrm{e}^{\mathrm{i}\omega^{(2-)}t}\right] \tag{S7}$$

A closer inspection of Eq. (S7) reveals that the up-converted transmitted pressure wave which propagated in the $\theta_{s,+1}$ direction with a frequency $\omega^{(1+)}$ is now received as a double-up converted signal at a frequency $\omega^{(2+)}$. Similarly, the down-converted signal in the $\mathbb{TX}$ mode which propagated in the $\theta_{s,-1}$ direction with a frequency $\omega^{(1-)}$ is received as a double-down converted signal at a frequency $\omega^{(2-)}$. This is while the fundamental transmitted wave component is received at the same original frequency (not-converted). As depicted in Fig. S2, the apparent frequency conversion which materializes in the STP phased array for the three mentioned channels—$\omega^{(1-)} \to \omega^{(2-)}$ from $\theta_{s,-1}$ direction, $\omega^{(1+)} \to \omega^{(2+)}$ from $\theta_{s,+1}$ direction and $\omega \to \omega$ from θ_s direction—allows a simple post-processing system to distinguish between the various channels in the $\mathbb{TX}$ and $\mathbb{RX}$ modes, thus enabling simultaneous multi-channel operations with two ports operating with out-of-band and one port operating with in-band communication potential. To demonstrate this, a simulation is carried out where multiple waves are incident from $\bar{\theta}_{s,-1} = -23.4°$, $\bar{\theta}_s = 0°$, and $\bar{\theta}_{s,+1} = 23.4°$, respectively at 990, 1000 and 1010 Hz using three individual line sources as shown in Fig. S2b. Figure S2a shows the FFT of the collected voltage signal output, where the dominant frequencies appear at 980 Hz, 1000 Hz, and 1020 Hz.

3 Supplemental Video File

The supplementary video file captures the operation of a conventional and an STP acoustic phased array. In order of appearance, the video shows:

- A schematic of a conventional phased array and an STP phased array with their respective $\mathbb{TX}$ and $\mathbb{RX}$ modes.
- Time-transient animation of a numerically simulated conventional array demonstrating a single transmission channel.
- Time-transient animation of a numerically simulated STP array demonstrating 5 different transmission channels (3 dominant).
- Directional breakdown and distribution of the FFT amplitudes at 980 Hz, 990 Hz, 1000 Hz, 1010 Hz, and 1020 Hz for conventional and STP arrays showing the principal transmission directions for both cases.
- Time-transient animation of a numerically simulated conventional array listening to signals from three directions. The conventional array exhibits negligible gain for waves arriving from directions other than its single principal direction.
- Time-transient animation of a numerically simulated STP array listening to signals from three principal directions at the same time. Considerable gain for all three directions is observed, and the STP array automatically up- or down-converts received signals based upon their direction of arrival.
